# The Influence of Geometry of Implants for Direct Skeletal Attachment of Limb Prosthesis on Rehabilitation Program and Stress-Shielding Intensity

**DOI:** 10.1155/2019/6067952

**Published:** 2019-07-08

**Authors:** Piotr Prochor, Eugeniusz Sajewicz

**Affiliations:** Department of Biocybernetics and Biomedical Engineering, Faculty of Mechanical Engineering, Bialystok University of Technology, Bialystok 15-351, Poland

## Abstract

The purpose of the research was to evaluate the influence of selected parameters of the implants for bone anchored prostheses on possibility of conducting static load bearing exercises and stress-shielding intensity. A press-fit implant, a threaded implant, and the proposed design were compared using the finite element method. For the analyses two features were examined: diameter (19.0 – 21.0 mm) and length (75.0 – 130.0 mm). To define the possibility of conducting rehabilitation exercises the micromotion of implants while axial loading with a force up to 1000 N was examined to evaluate the changes at implant-bone interface. The stress-shielding intensity was estimated by bone mass loss over 60 months. The results suggest that, in terms of micromotion generated during rehabilitation exercises, the threaded (max. micromotion of 16.00 *μ*m) and the proposed (max. micromotion of 45.43 *μ*m) implants ensure low and appropriate micromotion. In the case of the press-fit solution the load values should be selected with care, as there is a risk of losing primary stabilisation. The allowed forces (that do not stimulate the organism to generate fibrous tissue) were approx. 140 N in the case of the length of 75 mm, increasing up to 560 N, while using the length of 130 mm. Moreover, obtained stress-shielding intensities suggest that the proposed implant should provide appropriate secondary stability, similar to the threaded solution, due to the low bone mass loss during long-term use (improving at the same time more bone remodelling in distal Gruen zones, by providing lower bone mass loss by approx. 13% to 20% in dependency of the length and diameter used). On this basis it can be concluded that the proposed design can be an appropriate alternative to commercially used implants.

## 1. Introduction

Currently, interest in direct skeletal attachment (DSA) of limb prosthesis as a more functional solution than typical socket-suspended prostheses is constantly increasing. The use of implants for DSA allows avoiding the disadvantages resulting from the use of socket-suspended prostheses, such as skin irritations and abrasions, discomfort while sitting, or unnatural control over the prosthesis [1–5]. Unfortunately, these implants also have some unresolved shortcomings, like the possibility of infection at the site of penetration of the skin through the implant's percutaneous part of the fixation or a decrease in the quality of bone tissue around the implant, which may extort in its removal [6–8].

The possibility of achieving osseointegration depends on the correct implantation and on the effectiveness of the adopted rehabilitation process at the time of primary stabilisation [9–11]. Primary stabilisation can be defined as the stabilisation of the implant in the bone obtained after its implantation for the time of bone-healing process and bone tissues overgrowth over surface of the implant [12, 13]. Successively, during rehabilitation process, primary stabilisation is gradually replaced by secondary stabilisation, characterised by full connection of the implant within bone tissues providing its stable anchoring in the bone [14]. The rehabilitation process usually depends on static load bearing exercises (LBE), used among others in postimplantation conditions of the OPRA system (Osseointegrated Prostheses for the Rehabilitation of Amputees) [15, 16]. The simplest exercises rely on loading the head of the implant with the user's body mass, while the appropriate axial load value is obtained by using typical scales [17]. These activities are designed to stimulate bone tissue remodelling and to prepare bone for new biomechanical conditions [13, 18–21]. Lack of loading during healing period can slow down or even prevent achieving complete implant-bone connection [22]. Moreover, abnormal (e.g., excessive) loading or insufficient anchoring of the implant in bone tissues may lead to its micromotion [23–25]. According to Brunski, micromotion of just over 50 *μ*m can cause fibrous tissue formation at the implant-bone interface, preventing proper implant stability [26]. The influence of loads occurring during rehabilitation process in primary stabilisation on the obtained micromotion of the implant for DSA in bone tissue has not yet been analysed in the literature. The description of this phenomenon may allow for more appropriate selection of the rehabilitation process basing not only on individual patient parameters (e.g., bone tissue quality), but also on the features of implant construction (press-fit or threaded).

One of the latest papers analysing phenomena around these implants are Tomaszewski with others' articles [7, 8]. They proposed a modular implant using modern materials (glass-particle reinforced PEEK and Ti6Al4V), which is characterised by a small length of 75.0 mm, while other solutions reach up to 130.0 mm, e.g., Integral-Leg-Prosthesis (ILP) system [7, 8]. In their analyses, they included a typical implant diameter of 20.0 mm. The materials and construction parameters they used made it possible to reduce the stress-shielding effect in comparison to the ISP and the OPRA implants. However, it is still not known which factor has stronger impact in the reduction of stress-shielding intensity, material or overall dimensions of the implant. Comparison of the implants' functionality by considering both of the above-mentioned parameters would give objective and comparable results. Currently, such analyses are still not conducted.

Due to the problems related to the currently used implants for DSA, the authors proposed their own medical construction. The Limb Prosthesis Osseointegration Fixation System (LPOFS) was design to, among others, combine the advantages of the press-fit and the threaded implants as well as increase the probability of achieving proper primary and secondary osseointegration. These features are obtained by its modular construction, assuming the interaction of two parts, medullary and percutaneous. The medullary part, made of glass-particle reinforced PEEK, is a conical, triple-notched part, with spiral-shaped, rounded teeth on its outer surface. The percutaneous part is made of Ti6Al4V, with shaft that is penetrating soft tissues. The increased, in certain aspects, biomechanical functionality of the LPOFS in relation to standard solutions has been reported in previously published authors' papers [27, 28]. However, for further evaluation of the proposed implant, it is necessary to conduct analyses that would consider the influence of variable dimensions of its structure.

The aim of the presented paper was to determine the effect of the changes in two selected features of the implant for direct skeletal attachment: diameter (19.0 mm–21.0 mm) and length (75.0 mm–130.0 mm) on possibility of conducting static LBE in primary stabilisation and on stress-shielding intensity in secondary stabilisation. To conduct the research the finite element method was used, which is widely and successfully used in order to estimate problems such as bone remodelling or appropriate implant design and its configuration [29–31]. In the first part the authors simulated axial loading of the implants to evaluate the micromotion of the implants. In the second part the bone mass loss around the implants was evaluated with the use of the internal bone remodelling concept that considers a lazy zone [7, 8, 18, 19, 32]. The authors have analysed the typical press-fit and threaded implants and proposed by them the LPOFS [6, 27, 28]. The obtained results can be further used to determine the influence of the constructional features of the implants on the primary and secondary stabilisation.

## 2. Materials and Methods

### 2.1. CAD Models

**Figure 1:**
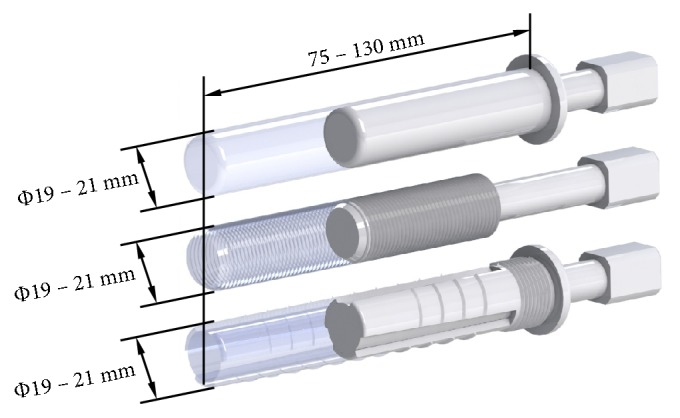
CAD models of analysed implants: from top: the press-fit implant, the threaded implant, and the LPOFS.

The first step in the presented research was the creation of the CAD models (Figure 1) with the use of SolidWorks 2016 software (Dassault Systèmes). In order to minimise the quantity of the factors affecting the obtained results the same overall dimensions of the implants were kept. A total of 36 implant-bone models were prepared, taking into account the variable length of implants from the range of 75 mm to 130 mm (with an increment of 5 mm) and a diameter from 19 mm to 21 mm (with an increment of 1 mm). Additionally, appropriate immersion in bone tissues of the threaded implant, reported in the literature, was also considered [33, 34]. The analysed constructional parameters correspond to the implants that were lately used in clinical conditions [7, 8]. Moreover, in the case of proposed implant as well as the press-fit solution, appropriate diametrical interference-fit value of 0.1 mm was included in the analyses, to reflect the implantation method. It was modelled by a geometrical difference between the diameter of the implant and the diameter of the reamed marrow cavity. In the threaded implant HA 5.0 shallow thread was considered. Its modifications are widely used as an anchoring element in the threaded medical implants. The thread's parameters are defined in appropriate ISO standard (ISO 5835:1991).

The authors used the left femur of an adult man (cut in half of its length to reflect the postamputation condition). The femur's overall dimensions were bone shaft diameter = 32.0 ± 2.0 mm, marrow cavity diameter = 16.0 ± 2.0 mm, and length from amputation level to the head of femur = 237.5 mm. The research conducted with the finite element method with the use of the same bone model for each of the implant design allowed to increase the objectivity of the results. It is achieved by analysing the same conditions for each considered setup, which would be nearly impossible in experimental research due to the fact that each bone differs in external (dimensional) or internal (bone density) features [35, 36].

### 2.2. FE Models

Created CAD models were exported to Ansys Workbench v16.2 software (Ansys, Inc.). Meshes were generated using Solid187 10-node tetrahedral elements with a maximum edge length of 3 mm. The mesh was gradually densified until further density changes influenced the results less than 2.5%. The models created consisted between 110,000 and 140,000 finite elements. Material properties used in the research are presented in Table 1.

### 2.3. Micromotion Influence on Implant-Bone Interface during Static Load Bearing Exercises

In order to simulate the static LBE, the implants heads were axially loaded with a force up to 1000 N (which relates to the patients with approx. mass up to 100 kg). The appropriate force values used by the patient during exercises are selected and allowed by a physician on the basis of the patient's individual data. However, the authors have considered an extreme case in which the patient loses stability during rehabilitation process and loads the implant with his total mass. To reflect primary stabilisation, appropriate coefficient of friction of 0.4 value was considered [39]. 

The authors analysed the sliding distance (achieved during axial loading), i.e., the displacement of the implant in bone tissue, which in the literature is described as micromotion of the implant. The maximum displacement of the implant above which bone tissues are stimulated to produce fibrous tissue was determined at 50 *μ*m, on the basis of the data presented by Brunski [26].

### 2.4. Stress-Shielding Intensity in Secondary Stabilisation

Previously created models were exported to Ansys Classic (Ansys Inc.) by creating batch files. In this case, appropriate anatomical loads were determined. The load values were set on the basis of the analyses of the OPRA system in experimental conditions [18, 19]. The same loading method was used by Tomaszewski and others during their analyses of internal bone remodelling [7, 8]. The first load case corresponds to a 25% gait cycle, the heel strike, and the other to a 55% gait cycle, toe-off (Table 2). Full osseointegration between the implant and the bone was considered to properly reflect secondary stabilisation. It was determined by sharing the same nodes by the finite elements of implants and bone tissues.

The stress-shielding intensity was determined as the estimation of internal bone remodelling around analysed implants. The process was taken into account by changing the density of the bone tissue during the use of the implants. The effect of the influence of bone density on bone mechanical properties (according to Carter and Hayes) was also considered [40]:

(1) $$\begin{array}{c} E=3790\rho^{3} \end{array}$$

**Figure 2:**
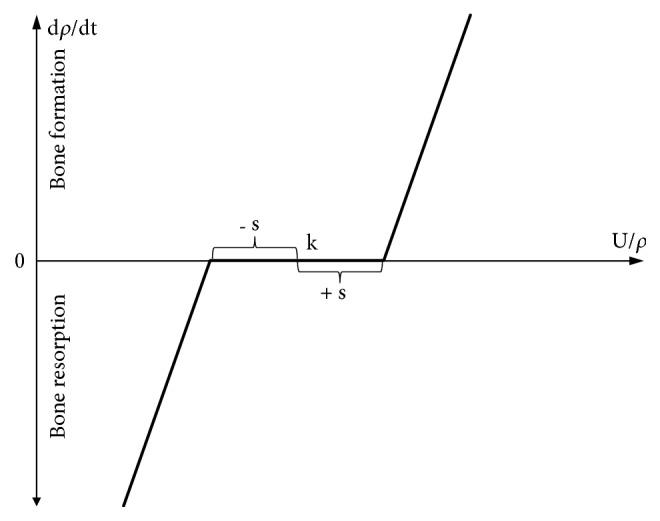
Bone remodelling approach used in analyses.

A widely used internal bone remodelling approach, which considers the lazy zone, was used for analyses [29]. Strain energy density was taken as bone remodelling stimulus. Considered bone remodelling concept is presented in Figure 2 and by formula (2). The constants' values were taken from the appropriate literature [32].

(2) dρdt=BUρ−1−skif  Uρ<1−sk0if  1−sk≤Uρ≤1+skBUρ−1+skif  Uρ>1+sk

Constant values in formula (2): B = 1.0 [(g/cm^3^)^2^/(MPa/CTU^−1^)]; s = 0.1; k = 0.004 [J/g]. Variable values in formula (2): U [J/cm^3^]; *ρ* [g/cm^3^].

In order to simulate continuous bone reconstruction around the implant, a suitable Fortran subroutine was used during the calculation process in which for each calculation step the density and Young's modulus of bone tissues were updated in each finite element. The initial values of bone Young's modulus and bone density presented in Table 1 were used. Poisson's coefficient was assumed to be constant for both bone tissues throughout the simulation. The threshold values for bone density were *ρ*_max_ = 2.000 g/cm^3^ and *ρ*_min_ = 0.200 g/cm^3^ [41].

**Figure 3:**
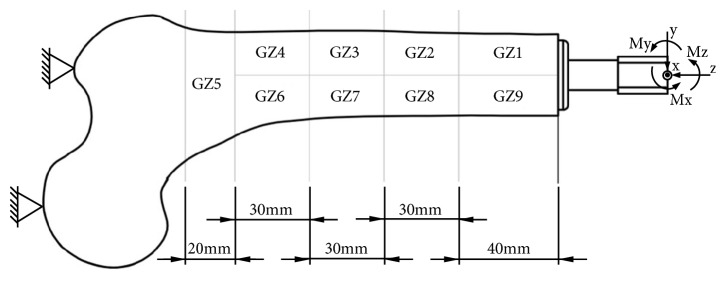
Frontal view of analysed left femur, analysed Gruen zones, and adopted coordinate system.

The obtained results are presented as changes in bone mass in individual bone areas (Figure 3), which are similar to Gruen zones, distinguished for hip stems [42]. The same bone remodelling algorithm was used in authors' previous paper in order to estimate the bone density changes after 60 months of using each of analysed implants [43]. The number of loops to be used was determined in the above-mentioned paper, on the basis of the OPRA system analyses and comparative analysis with the clinical results obtained by Xu and Robinson [9]. The described methodology shows similarities with the methodology of Tomaszewski and others, who presented its correctness [7, 8].

**Figure 4:**
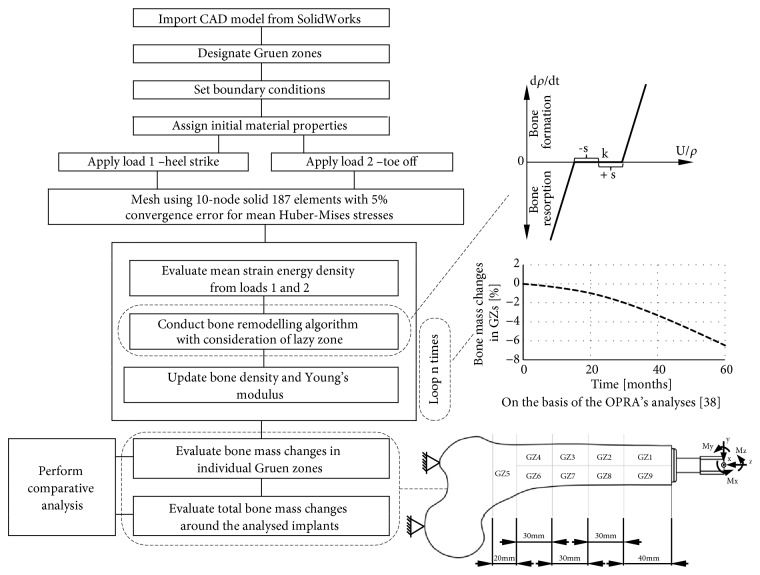
Scheme of conducted analyses process.

The schematic analysis process was presented in Figure 4.

## 3. Results

### 3.1. Micromotion Generated by Static Load Bearing Exercises

**Figure 5:**
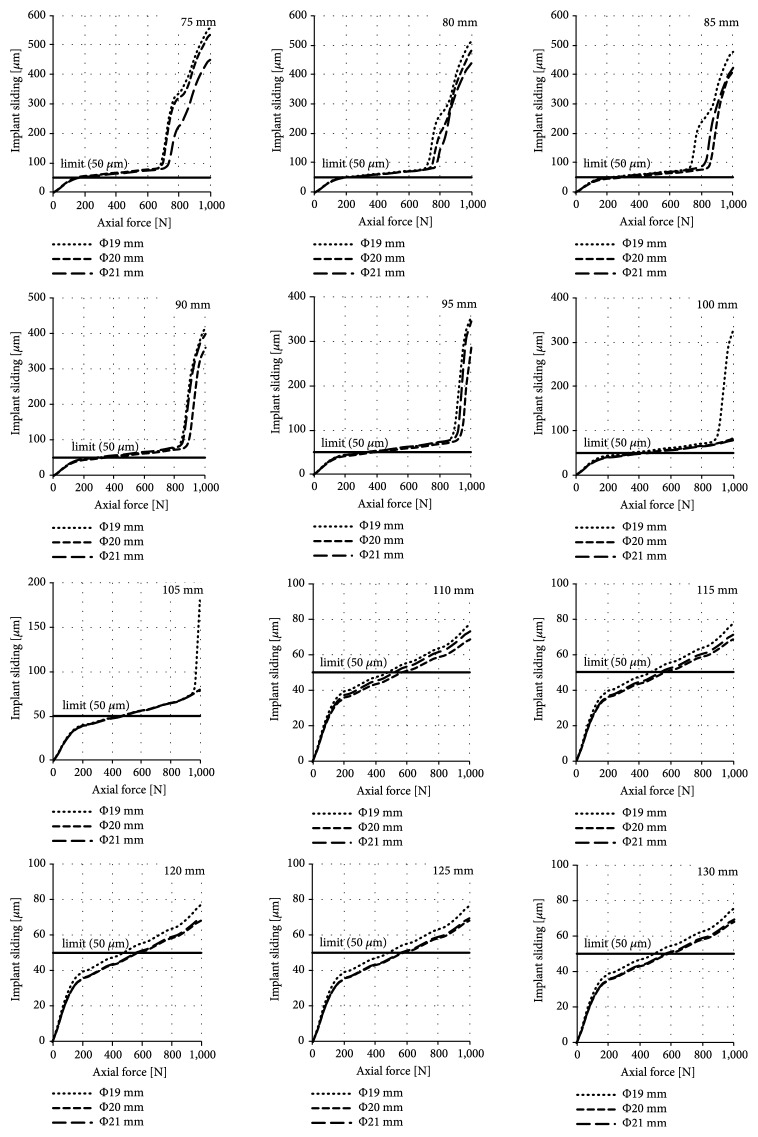
Sliding distance occurring during static LBE for the press-fit implant.

**Figure 6:**
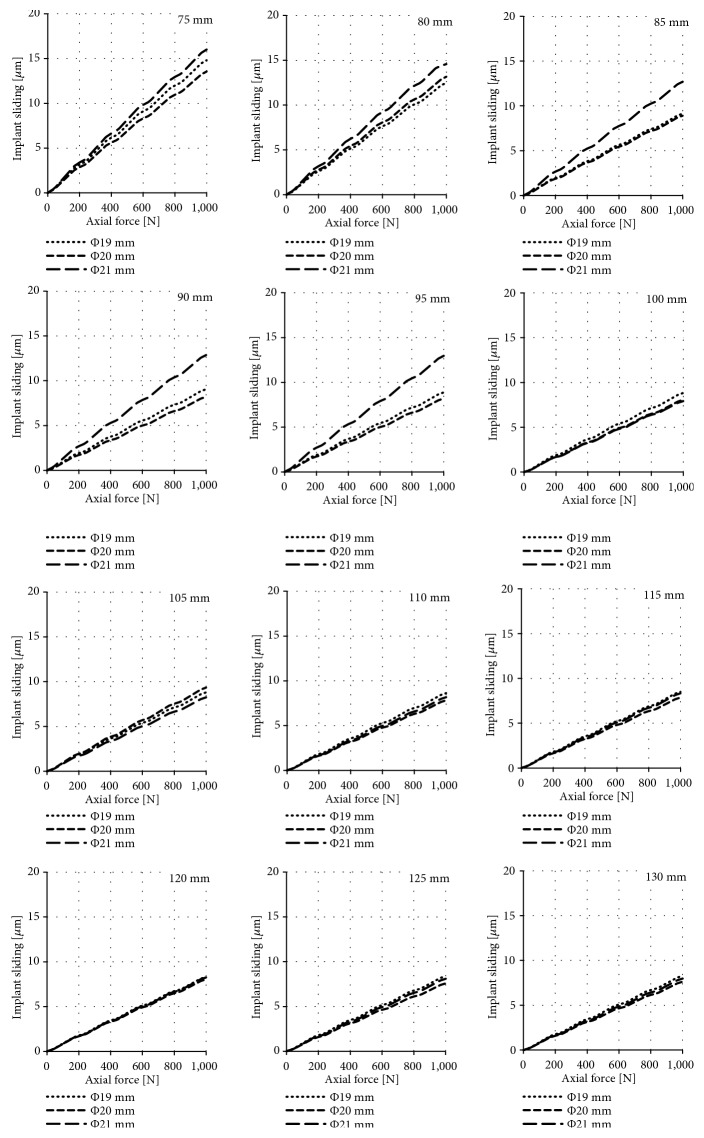
Sliding distance occurring during static LBE for the threaded implant.

**Figure 7:**
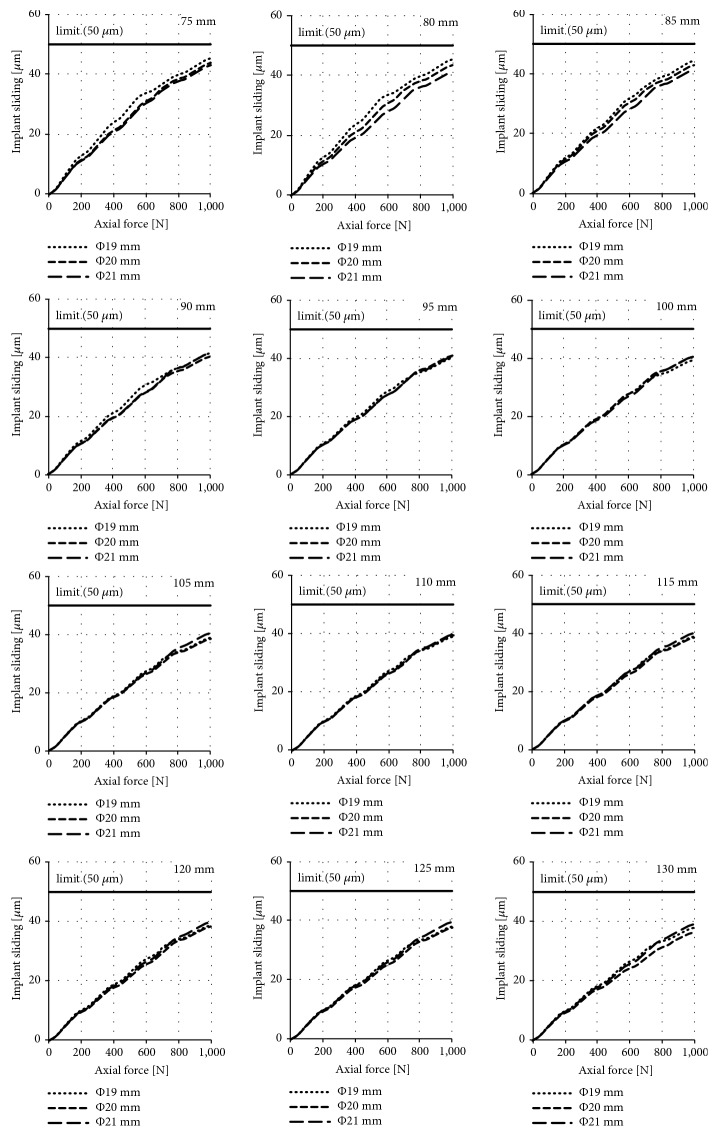
Sliding distance occurring during static LBE for the LPOFS.

The results of the obtained sliding distance values for individual diameters and lengths of implants during their axial loading are presented in Figures 5, 6, and 7 for the press-fit implant, the threaded implant, and the LPOFS, respectively.

**Figure 8:**
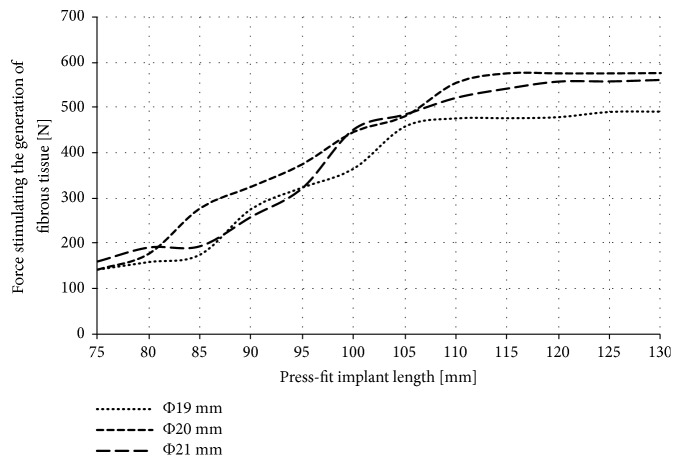
Force stimulating the generation of fibrous tissue for the press-fit implant.

Only in the case of the press-fit implant, the obtained forces caused the implant sliding of at least 50 *μ*m. Force stimulating the generation of fibrous tissue for the press-fit implant is presented in Figure 8.

### 3.2. Long-Term Bone Mass Change

**Figure 9:**
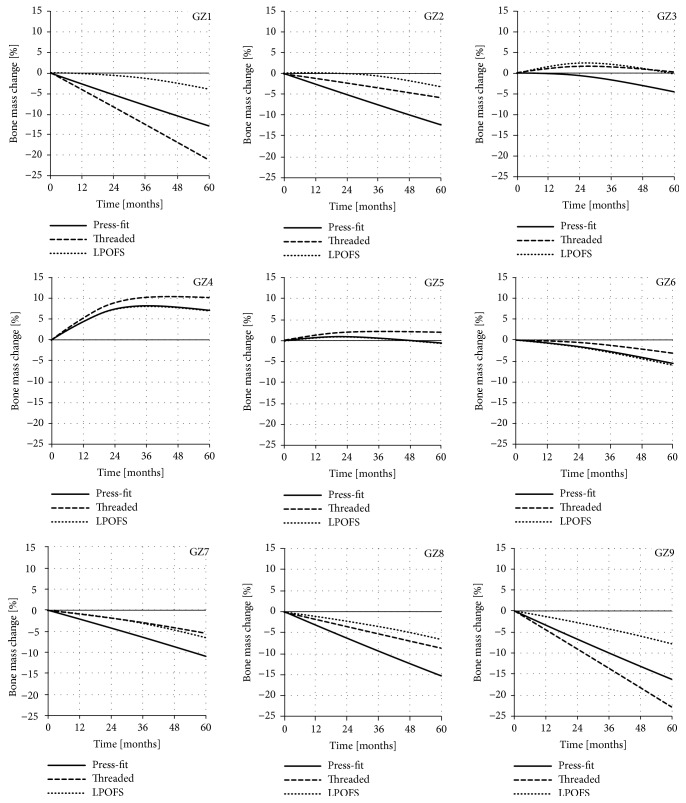
The changes in bone mass in each of the analysed Gruen zones for implants length of 100 mm and diameter of 20 mm during 60 months of use.

It is estimated that, after 60 months from full osseointegration and proper loading of the implant, the bone adaptation process stabilises [44]. For this reason, the changes in bone mass in each analysed zone were analysed for this period of time. The exemplary results in individual Gruen zones, obtained for the implants length of 100 mm and diameter of 20 mm, are presented in Figure 9.

**Figure 10:**
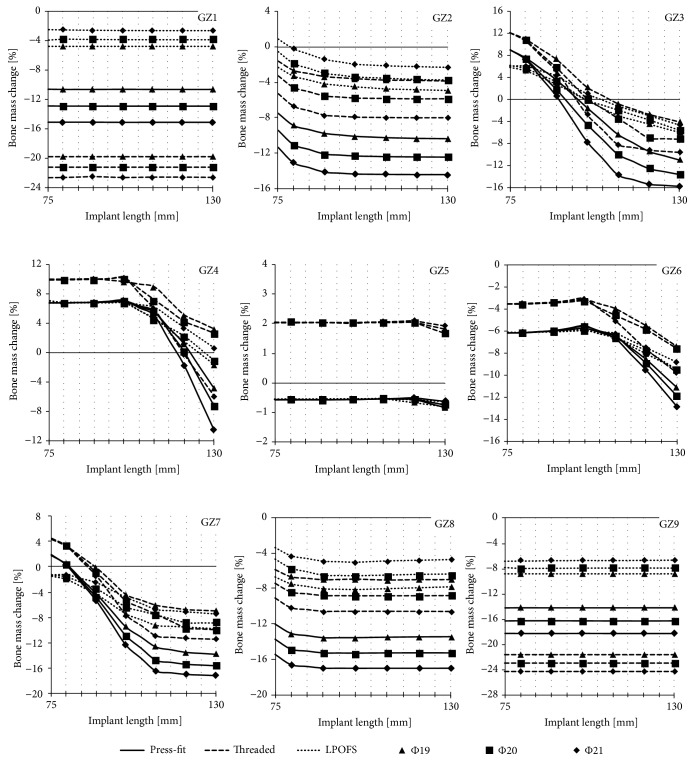
The changes in bone mass in each of the analysed Gruen zones after 60 months of implant loading.

As it can be noted, the data collected consists of series of results for a single analysis of a given length and diameter of implants. In order to present majority of the results, it was decided to limit them only to bone mass change in individual Gruen zones (GZ) after 60 months of implants' use, i.e., after the bone remodelling process stabilises [44]. Such processed results are presented in Figure 10.

### 3.3. Results Summary

Tables 3, 4, and 5 present the summary of the data obtained during the research that is micromotion during SLBE in primary stabilisation as well as bone mass changes after 60 months of secondary stabilisation.

## 4. Discussion

The effectiveness of static LBE might depend on the micromotion of the implant in bone tissues, which significantly depends on its shape and length [9–11]. This motion may prevent obtaining appropriate stabilisation of the implant [22]. The description of the micromotion achieved during the static LBE while using particular type of the implant, taking into account its variable general dimensions, may lead to obtaining more effective rehabilitation plans for individual patients.

The reduction of stress-shielding effect is possible by taking into account the use of modern engineering materials, such as PEEK, or by modifying the overall dimensions of the implant, like its length. However, it is important to determine which feature has caused a significant reduction in stress-shielding intensity and whether using a different implant, changing only its length, will result in a similar reduction of stress-shielding intensity.

### 4.1. Possibility of Conducting Static Load Bearing Exercises in Primary Stabilisation

While analysing the micromotion caused during the axial loading of the implants there can be noted an intensive influence of the length and the shape of the implant (in all analysed implants' types) on the obtained results. However, no significant effect of the diameter of the threaded implant and the LPOFS was noticed. In the case of the press-fit implant, the use of a diameter of 21 mm can increase the force stimulating the generation of fibrous tissue by about 100 N in relation to the diameter of 19 mm in majority of analysed lengths.

The highest micromotion was obtained while using the press-fit implant; in the case of short length of 75 mm, the force stimulating the generation of fibrous tissue was only about 150 N. Increasing the length to 115 mm can increase this force to about 570 N. Further increase of the length did not change the anchoring effectiveness of the implant in bone tissues, already obtained with length of 115 mm. This suggests that, in terms of the analysed feature, typical press-fit designs should have appropriate lengths to enable the possibility of applying static LBE in primary stabilisation. Moreover, in every analysed dimension of the press-fit implant, there was a force that caused the micromotion of the implant over 50 *μ*m. What is more, while using the length up to 100 mm, there were certain forces, above which the micromotion was suddenly and vastly increasing. On this basis it can be stated that there exist forces that can lead to a complete loss of primal connection between bone cells and the surface of the implant. For this reason, the selection of appropriate loading of the implant with patient's own mass during static LBE should be selected with particular care, considering general dimensions of the implant in use.

The results obtained for the threaded implants present linear dependencies. The progressive increase in the axial force also causes a progressive increase in implant micromotion. The maximum micromotion was 15 *μ*m and 8 *μ*m, respectively when using a short (75 mm) and long (130 mm) structure, assuming maximal force of 1000 N. These displacements are significantly lower than displacements inducing fibrous tissue formation. Therefore, it can be assumed that the use of the threaded implant allows for safe loading during LBE, regardless of the implant general dimensions.

While analysing the micromotion of the LPOFS during its axial loading, similar linear dependencies as in the case of the threaded implant were obtained. Additional similarity was the fact that the increase in the length of the LPOFS does not significantly increase its anchoring efficiency in bone tissues. For this reason, a short version should be used as it has the same biomechanical effectiveness in primary stabilisation as its longer design. However, the difference between the LPOFS and the threaded implant was the obtained values of its micromotion, which was reaching the values of approx. 40 *μ*m to 45 *μ*m, while using maximal force of 1000 N. These micromotions are close to values stimulating the generation of fibrous tissue. For this reason, if the implantation system proposed by the authors would be used in clinical conditions, extreme loads should be avoided during static LBE.

### 4.2. Stress-Shielding Intensity in Secondary Stabilisation

In opposition to the first part of the study, the diameter of the implant had significant influence on the obtained results of stress-shielding intensity in individual Gruen zones. This suggests that, due to the long-term use of the implant, its proper diameter should be considered and appropriately selected.

As expected, the reduction of bone mass in GZ1 and GZ9 is constant for all analysed lengths of implants, as the smallest implant length (75 mm) greatly exceeds the length of these areas. In the case of these Gruen zones, significant differences in the intensity of stress-shielding intensity when using different shape of the implants are also noticeable. For the press-fit implant there was a constant bone mass loss of -10.5%, -12.8%, -15.0% (GZ1) and -14.2%, -16.2%, -18.2% (GZ9) for diameters 19 mm, 20 mm, and 21 mm, respectively. The highest bone mass loss was obtained during the use of the threaded implant. The loss was -19.7%, -21.2%, -22.5% (GZ1) and -21.6%, -30.0%, -24.2% (GZ9), also for diameters of 19 mm, 20 mm, and 21 mm. Thus, the tendency that the use of a smaller implant diameter reduces the intensity of stress-shielding was maintained. This trend was broken in the case of the results obtained for the LPOFS; the lowest bone mass loss in distal Gruen zones was obtained for the implant diameter of 21 mm, which, regardless of the length of the implant, was about -2.6% (GZ1) and -6.6% (GZ9). Slightly higher loss (-3.8% for GZ1 and -7.8% for GZ9) was obtained for a diameter of 20 mm, while the highest bone mass loss (-4.7% for GZ1 and -8.8% for GZ9) was obtained for the smallest diameter of 19 mm.

Another notable feature is constant small bone mass changes in GZ5 in all analysed shapes and general dimensions of the implants. The bone mass loss values obtained when using the press-fit implant and the LPOFS were less than -1%, while in the case of the threaded implant, these changes were approx. +2%. This difference might be a cause of the initial immersion in the bone tissues of the threaded implant, generating a significant intensification of stress-shielding in distal Gruen zones (GZ1 and GZ9). This results in a concentration of stresses in the smaller area of the bone in relation to the press-fit implant and the LPOFS. Moreover, in GZ5, where the implants' lengths end before this zone starts, the increase of bone mass loss does not occur until the length is greater than 110 mm for the press-fit implant and the LPOFS. In the case of the threaded implant, the intensity of stress-shielding in GZ5 increases after the length of 120 mm. However, the intensity increases only up to -0.5%.

The results obtained for GZ2 and GZ8 present increasing stress-shielding intensity with the increase of the length of the implants up to 100 mm. Above this length, there was no further intensification of bone mass loss. In opposition to more distal Gruen zones (GZ1 and GZ9) in GZ2 and GZ8, the highest stress-shielding was obtained while using the press-fit implant (from -10.3% to 14.4% in GZ2 and from -13.4% and -17.0% in GZ8). For the two remaining implants, the intensity of stress-shielding was similar with slightly lower bone mass changes for the LPOFS (from +0.9% up to -8% in GZ2 and from -3.3% up to -10% in GZ8). The effect of the diameter of implants on the obtained results was similar to the results for GZ1 and GZ9.

The highest influence of the implant length on the obtained results occurred in GZ3, GZ4, GZ6, and GZ7. In the case of GZ3 and GZ7, the increase of length had a constant effect on the intensity of stress-shielding. Increasing the length from 75 mm to 130 mm can change bone mass even up to 24%. It is worth noticing that small lengths of implants were causing the increase of bone mass. In opposition to previously described Gruen zones (that is, GZ1, GZ2, GZ5, GZ8, and GZ9), in GZ3 and GZ7 the threaded implant allowed the highest reduction of stress-shielding. In two remaining implant types, for the length up to 90 mm, the intensity of stress-shielding in GZ3 and GZ7 was lower for the press-fit implant. Above this length, the bone mass loss was lower for the LPOFS.

The intensity of stress-shielding in GZ4 and GZ6 again was the lowest while using the threaded solution. The press-fit solution ensured the same bone mass loss as the LPOFS while using the same lengths of the implants. The bone mass loss was constant until 100 mm of implant's length (for all analysed implant types); with the use of longer solutions, the stress-shielding intensity was increasing linearly. In this case, the diameter used did not influence the obtained results.

The results obtained in GZ4, GZ5, and GZ6 are particularly important due to the risk of periprosthetic fracture in the proximal parts of the bone. During the long-term use of implants, mechanical properties of bone tissue are changing, i.e., due to changes in bone density. As a result, it can lead to weakening of the bone due to the reduction of its density. While loading the head of the implant, characterised by relatively high stiffness, the loads are transferred, in the case of implants for direct skeletal attachment of limb prosthesis, to the proximal area of the bone. Inability to provide the appropriate biological signal that stimulates bone remodelling, which in terms of biomechanics takes a form of correct load transfer throughout the whole bone, may therefore increase the risk of periprosthetic fracture. The results obtained by the authors allow estimating the influence of the analysed factors on the possibility of occurrence of above-mentioned bone fracture.

It should be noted that the obtained results are a computer simulation of bone tissue remodelling. However, the process was adapted to clinical bone remodelling evaluations, available in the literature [9]. Moreover, the tendencies obtained for the press-fit and threaded implants of 100 mm length present similarities to the results obtained by Tomaszewski with others [7, 8].

## 5. Limitations of the Study

The use of the finite element method required adapting some simplifications, which resulted in limitations of the presented study. The major one is the use of isotropic mechanical properties of bone tissues and considering their homogeneity as input values. However, the use of patient's specific bone internal features would introduce additional variables that could make the results less common. More limitations result from considering specified loading method that can vary significantly if different prosthetic components are used or different patient-specific loads are examined [4, 5]. Moreover, the data is also affected by straight insertion of implants in the bone shaft, while clinically it is also often placed at an angle, making Gruen zones asymmetrical. Nevertheless, the aim of the research was to conduct the comparative analysis of selected implants in controlled in silico environment, which should allow for objectively evaluation of considered implants.

### 5.1. Future Studies

The research can be further developed with consideration of different type of prosthesis' components, which have significant influence on the loads generated on the head of the implant [4, 5]. In the future the authors consider conducting in vitro research in order to confirm the functionality of the presented implant. With the successful data, the research could be expanded by in vivo tests with the use of animal subject.

## 6. Conclusions

On the basis of obtained data, it can be concluded that the threaded solution should allow obtaining the lowest micromotion during static LBE. In the case of proposed implant, the values of obtained micromotion suggest that it also might allow conducting rehabilitation exercises. While using the press-fit solution, the loads should be selected with certain care as there is a risk of creating micromotion that stimulates the generation of fibrous tissue and losing the chance of obtaining appropriate primary stability. Moreover, the obtained results suggest that the use of modular implant, with the medullar part that is characterised by relatively low stiffness, in total ensures the lowest intensity of stress-shielding for most of analysed cases. However, the threaded solution ensured similar long-term biomechanical functionality as modular design, with the difference of high bone mass loss in distant Gruen zones. On this basis it can be concluded that using modular implant can positively influence the rehabilitation program in primary stability as well as bone remodelling in secondary stability, which was confirmed with the data obtained for the implant proposed by the authors.

## Figures and Tables

**Table 1:** Mechanical properties of materials used for analyses.

Property	Material			
	PEEK (glass-particle reinforced) [7, 8]	Ti6Al4V [37]	Cortical bone [38]	Cancellous bone [38]
Young's modulus [GPa]	12.50	110.00	20.00	0.96
Density [g/cm^3^]	1.320	4.500	1.740	0.630
Poisson's ratio	0.40	0.33	0.30	0.12

**Table 2:** Loading cases [7, 8, 18, 19].

	F_x_ [N]	F_y_ [N]	F_z_ [N]	M_x_ [Nm]	M_y_ [Nm]	M_z_ [Nm]
Heel strike	100.0	-20.0	780.0	30.8	-7.2	-2.0
Toe-off	120.0	40.0	180.0	37.3	4.1	0.0

**(a):** 

Diameter [mm]		Implant type	Length [mm]											
			75	80	85	90	95	100	105	110	115	120	125	130
Φ 19	Implant sliding under a force of 1000 N	Press-fit	*557.38*	*511.78*	*478.15*	*414.31*	*355.50*	*327.29*	*180.78*	**77.68**	**77.58**	**76.88**	**76.20**	**76.02**
		Threaded	14.81	14.58	9.13	9.06	8.88	8.83	8.80	8.63	8.51	8.30	8.39	8.28
		LPOFS	45.42	45.43	44.45	41.53	40.16	39.39	39.12	38.89	38.79	38.76	38.17	37.79

Italic: over 100 *µ*m; bold: 50 *µ*m to 100 *µ*m; underline: 25 *µ*m to 50 *µ*m; normal-regular: to 25 *µ*m.

**(b):** 

Diameter [mm]	Bone mass change after 60 months	Implant type	Length [mm]											
			75	80	85	90	95	100	105	110	115	120	125	130
Φ 19	G1	Press-fit	**-10.57**	**-10.59**	**-10.59**	**-10.60**	**-10.59**	**-10.58**	**-10.58**	**-10.59**	**-10.59**	**-10.60**	**-10.60**	**-10.59**
		Threaded	*-19.74*	*-19.74*	*-19.74*	*-19.74*	*-19.74*	*-19.74*	*-19.74*	*-19.75*	*-19.75*	*-19.75*	*-19.76*	*-19.76*
		LPOFS	-4.70	-4.72	-4.72	-4.73	-4.74	-4.75	-4.77	-4.79	-4.79	-4.79	-4.79	-4.79
	G2	Press-fit	***-7.51***	***-8.90***	***-9.35***	***-9.79***	***-9.96***	**-10.12**	**-10.19**	**-10.27**	**-10.31**	**-10.35**	**-10.37**	**-10.38**
		Threaded	-1.57	-2.65	-3.00	-3.35	-3.47	-3.59	-3.67	-3.75	-3.77	-3.78	-3.82	-3.86
		LPOFS	-2.27	-3.28	-3.72	-4.16	-4.33	-4.50	-4.62	-4.74	-4.78	-4.83	-4.87	-4.92
	G3	Press-fit	8.99	7.60	5.60	3.61	1.06	-1.50	-3.91	***-6.33***	***-7.86***	***-9.39***	**-10.18**	**-10.96**
		Threaded	12.11	10.97	9.15	7.33	4.77	2.21	0.72	-0.78	-1.74	-2.70	-3.38	-4.07
		LPOFS	5.77	5.19	3.97	2.76	1.27	-0.21	-1.58	-2.95	-3.70	-4.45	***-5.20***	***-5.96***
	G4	Press-fit	6.76	6.76	6.79	6.82	6.86	6.89	6.21	5.52	3.37	1.22	-1.79	-4.80
		Threaded	9.90	9.91	9.93	9.95	9.77	9.58	9.23	8.88	6.97	5.07	4.13	3.19
		LPOFS	6.76	6.76	6.79	6.81	6.79	6.77	5.73	4.68	3.11	1.54	-0.03	-1.60
	G5	Press-fit	-0.57	-0.59	-0.58	-0.58	-0.58	-0.57	-0.56	-0.55	-0.56	-0.57	-0.70	-0.84
		Threaded	2.05	2.05	2.05	2.05	2.03	2.01	2.04	2.07	2.06	2.05	1.94	1.83
		LPOFS	-0.57	-0.57	-0.57	-0.56	-0.56	-0.56	-0.56	-0.56	-0.62	-0.69	-0.75	-0.82
	G6	Press-fit	***-6.20***	***-6.19***	***-6.11***	***-6.02***	***-5.90***	***-5.78***	***-6.13***	***-6.48***	***-7.54***	***-8.59***	***-9.87***	**-11.15**
		Threaded	-3.55	-3.55	-3.50	-3.46	-3.31	-3.16	-3.57	-3.98	-4.76	***-5.55***	***-6.50***	***-7.45***
		LPOFS	***-6.19***	***-6.17***	***-6.13***	***-6.08***	***-6.02***	***-5.96***	***-6.32***	***-6.67***	***-7.39***	***-8.10***	***-8.82***	***-9.53***
	G7	Press-fit	1.78	0.41	-1.92	-4.25	***-6.89***	***-9.54***	**-11.11**	**-12.68**	**-13.10**	**-13.53**	**-13.67**	**-13.82**
		Threaded	4.33	3.27	1.55	-0.18	-2.33	-4.49	***-5.33***	***-6.16***	***-6.50***	***-6.83***	***-6.93***	***-7.02***
		LPOFS	-1.55	-2.05	-3.23	-4.40	***-5.97***	***-7.53***	***-8.42***	***-9.30***	***-9.43***	***-9.56***	***-9.68***	***-9.81***
	G8	Press-fit	**-12.02**	**-13.07**	**-13.31**	**-13.55**	**-13.55**	**-13.55**	**-13.52**	**-13.50**	**-13.48**	**-13.46**	**-13.45**	**-13.44**
		Threaded	***-5.83***	***-6.64***	***-6.80***	***-6.97***	***-6.93***	***-6.89***	***-6.95***	***-7.00***	***-6.98***	***-6.95***	***-6.95***	***-6.95***
		LPOFS	***-6.66***	***-7.47***	***-7.76***	***-8.04***	***-8.08***	***-8.13***	***-8.07***	***-8.02***	***-7.97***	***-7.91***	***-7.86***	***-7.80***
	G9	Press-fit	**-14.20**	**-14.19**	**-14.19**	**-14.19**	**-14.19**	**-14.19**	**-14.19**	**-14.19**	**-14.19**	**-14.19**	**-14.19**	**-14.19**
		Threaded	*-21.63*	*-21.60*	*-21.60*	*-21.61*	*-21.63*	*-21.64*	*-21.63*	*-21.62*	*-21.61*	*-21.61*	*-21.63*	*-21.64*
		LPOFS	***-8.88***	***-8.87***	***-8.86***	***-8.86***	***-8.86***	***-8.86***	***-8.87***	***-8.87***	***-8.87***	***-8.86***	***-8.85***	***-8.84***

Italic: over -15%; bold: -10% to -15%; bold-italic: -5% to -10%; underline: 0% to -5%; normal-regular: bone mass increase.

**(a):** 

Diameter [mm]		Implant type	Length [mm]											
			75	80	85	90	95	100	105	110	115	120	125	130
Φ 20	Implant sliding under a force of 1000 N	Press-fit	*535.48*	*480.14*	*409.95*	*358.93*	*287.23*	**83.15**	**79.87**	**68.37**	**68.24**	**68.27**	**68.28**	**68.26**
		Threaded	13.55	13.16	8.91	8.19	8.20	8.07	9.35	7.83	7.87	8.04	7.59	7.62
		LPOFS	44.01	43.40	42.80	40.31	40.82	40.49	38.42	39.29	38.32	38.45	37.69	36.43

Italic: over 100 *µ*m; bold: 50 *µ*m to 100 *µ*m; underline: 25 *µ*m to 50 *µ*m; normal-regular: to 25 *µ*m.

**(b):** 

Diameter [mm]	Bone mass change after 60 months	Implant type	Length [mm]											
			75	80	85	90	95	100	105	110	115	120	125	130
Φ 20	G1	Press-fit	**-12.84**	**-12.86**	**-12.86**	**-12.86**	**-12.86**	**-12.86**	**-12.87**	**-12.87**	**-12.87**	**-12.87**	**-12.87**	**-12.87**
		Threaded	*-21.15*	*-21.17*	*-21.17*	*-21.17*	*-21.18*	*-21.19*	*-21.19*	*-21.19*	*-21.18*	*-21.18*	*-21.18*	*-21.17*
		LPOFS	-3.83	-3.72	-3.73	-3.75	-3.76	-3.78	-3.78	-3.78	-3.78	-3.79	-3.79	-3.78
	G2	Press-fit	***-9.44***	**-11.07**	**-11.60**	**-12.13**	**-12.24**	**-12.35**	**-12.38**	**-12.42**	**-12.43**	**-12.45**	**-12.46**	**-12.47**
		Threaded	-3.28	-4.60	***-5.07***	***-5.55***	***-5.67***	***-5.79***	***-5.81***	***-5.84***	***-5.86***	***-5.88***	***-5.87***	***-5.86***
		LPOFS	-0.48	-1.82	-2.37	-2.92	-3.13	-3.34	-3.41	-3.48	-3.55	-3.63	-3.65	-3.68
	G3	Press-fit	9.02	7.41	4.63	1.85	-1.35	-4.54	***-7.24***	***-9.94***	**-11.23**	**-12.52**	**-13.05**	**-13.58**
		Threaded	12.17	10.80	8.34	5.89	3.05	0.21	-1.57	-3.36	***-5.14***	***-6.92***	***-7.03***	***-7.14***
		LPOFS	6.21	5.64	4.45	3.26	1.58	-0.10	-0.98	-1.87	-2.75	-3.64	-4.59	***-5.54***
	G4	Press-fit	6.75	6.76	6.80	6.85	6.95	7.05	6.36	5.67	2.93	0.19	-3.49	***-7.17***
		Threaded	9.89	9.90	9.93	9.96	10.02	10.08	8.60	7.13	5.66	4.18	3.38	2.58
		LPOFS	7.02	6.76	6.77	6.78	6.81	6.85	5.70	4.55	3.40	2.26	0.61	-1.03
	G5	Press-fit	-0.58	-0.57	-0.58	-0.58	-0.58	-0.57	-0.56	-0.55	-0.55	-0.55	-0.65	-0.75
		Threaded	2.05	2.05	2.05	2.04	2.04	2.04	2.04	2.04	2.05	2.05	1.86	1.67
		LPOFS	-0.57	-0.56	-0.57	-0.58	-0.57	-0.57	-0.57	-0.56	-0.56	-0.56	-0.65	-0.75
	G6	Press-fit	***-6.20***	***-6.18***	***-6.08***	***-5.99***	***-5.80***	***-5.61***	***-6.03***	***-6.45***	***-7.70***	***-8.94***	**-10.42**	**-11.91**
		Threaded	-3.56	-3.56	-3.51	-3.45	-3.37	-3.29	-3.94	-4.58	***-5.23***	***-5.87***	***-6.75***	***-7.63***
		LPOFS	***-6.14***	***-6.17***	***-6.13***	***-6.08***	***-6.01***	***-5.95***	***-6.28***	***-6.62***	***-7.20***	***-7.79***	***-8.65***	***-9.52***
	G7	Press-fit	1.87	0.33	-2.23	-4.80	***-7.86***	**-10.93**	**-12.86**	**-14.78**	*-15.09*	*-15.40*	*-15.52*	*-15.63*
		Threaded	4.43	3.27	1.13	-1.01	-3.25	***-5.49***	***-6.56***	***-7.62***	***-8.69***	***-9.75***	***-9.88***	**-10.02**
		LPOFS	-1.39	-1.66	-2.58	-3.51	-4.95	***-6.39***	***-6.99***	***-7.60***	***-8.20***	***-8.81***	***-8.82***	***-8.83***
	G8	Press-fit	**-13.73**	**-14.85**	*-15.06*	*-15.27*	*-15.28*	*-15.29*	*-15.28*	*-15.27*	*-15.26*	*-15.26*	*-15.26*	*-15.26*
		Threaded	***-7.41***	***-8.38***	***-8.58***	***-8.77***	***-8.78***	***-8.79***	***-8.79***	***-8.79***	***-8.79***	***-8.79***	***-8.78***	***-8.77***
		LPOFS	-4.68	***-5.74***	***-6.15***	***-6.56***	***-6.59***	***-6.62***	***-6.57***	***-6.53***	***-6.49***	***-6.45***	***-6.43***	***-6.41***
	G9	Press-fit	*-16.27*	*-16.24*	*-16.24*	*-16.24*	*-16.25*	*-16.25*	*-16.26*	*-16.27*	*-16.27*	*-16.27*	*-16.26*	*-16.26*
		Threaded	*-22.97*	*-22.99*	*-22.97*	*-22.96*	*-22.95*	*-22.94*	*-22.95*	*-22.95*	*-22.96*	*-22.96*	*-22.97*	*-22.98*
		LPOFS	***-7.79***	***-7.91***	***-7.90***	***-7.90***	***-7.88***	***-7.87***	***-7.87***	***-7.86***	***-7.86***	***-7.86***	***-7.86***	***-7.87***

Italic: over -15%; bold: -10% to -15%; bold-italic: -5% to -10%; underline: 0% to -5%; normal-regular: bone mass increase.

**(a):** 

Diameter [mm]		Implant type	Length [mm]											
			75	80	85	90	95	100	105	110	115	120	125	130
Φ 21	Implant sliding under a force of 1000 N	Press-fit	*448.33*	*438.29*	*423.89*	*399.38*	*346.58*	**78.66**	**78.66**	**72.96**	**70.74**	**69.60**	**69.55**	**69.03**
		Threaded	16.00	12.49	12.69	12.87	12.96	7.92	8.25	8.18	8.35	8.24	8.10	7.98
		LPOFS	42.98	41.22	41.22	41.44	40.51	40.49	40.49	39.85	39.98	40.06	39.55	39.07

Italic: over 100 *µ*m; bold: 50 *µ*m to 100 *µ*m; underline: 25 *µ*m to 50 *µ*m; normal-regular: to 25 *µ*m.

**(b):** 

Diameter [mm]	Bone mass change after 60 months	Implant type	Length [mm]											
			75	80	85	90	95	100	105	110	115	120	125	130
Φ 21	G1	Press-fit	*-15.05*	*-15.05*	*-15.05*	*-15.05*	*-15.06*	*-15.07*	*-15.07*	*-15.07*	*-15.06*	*-15.06*	*-15.06*	*-15.07*
		Threaded	*-22.55*	*-22.54*	*-22.53*	*-22.51*	*-22.53*	*-22.54*	*-22.54*	*-22.53*	*-22.53*	*-22.53*	*-22.53*	*-22.54*
		LPOFS	-2.46	-2.50	-2.52	-2.54	-2.56	-2.58	-2.58	-2.59	-2.59	-2.59	-2.59	-2.59
	G2	Press-fit	**-11.37**	**-13.12**	**-13.65**	**-14.19**	**-14.29**	**-14.39**	**-14.41**	**-14.43**	**-14.44**	**-14.45**	**-14.45**	**-14.45**
		Threaded	***-5.24***	***-6.69***	***-7.21***	***-7.73***	***-7.84***	***-7.95***	***-7.97***	***-7.99***	***-8.01***	***-8.02***	***-8.02***	***-8.01***
		LPOFS	0.87	-0.22	-0.80	-1.39	-1.66	-1.92	-2.01	-2.09	-2.14	-2.18	-2.22	-2.27
	G3	Press-fit	9.05	7.27	3.96	0.66	-3.51	***-7.67***	**-10.67**	**-13.68**	**-14.51**	*-15.34*	*-15.53*	*-15.72*
		Threaded	12.21	10.72	7.99	5.27	1.24	-2.79	***-5.51***	***-8.23***	***-8.74***	***-9.25***	***-9.45***	***-9.65***
		LPOFS	5.98	6.01	5.14	4.27	2.64	1.01	-0.08	-1.16	-2.01	-2.86	-3.71	-4.56
	G4	Press-fit	6.74	6.76	6.80	6.85	6.98	7.11	6.10	5.08	1.72	-1.64	***-6.03***	**-10.43**
		Threaded	9.89	9.91	9.95	9.99	10.08	10.17	8.05	5.93	2.84	-0.25	-3.07	***-5.88***
		LPOFS	6.76	6.76	6.77	6.78	6.79	6.79	6.45	6.11	4.73	3.35	1.97	0.59
	G5	Press-fit	-0.59	-0.57	-0.58	-0.58	-0.58	-0.57	-0.56	-0.55	-0.53	-0.52	-0.58	-0.63
		Threaded	2.04	2.03	2.04	2.04	2.04	2.05	2.06	2.07	2.09	2.10	2.02	1.93
		LPOFS	-0.57	-0.57	-0.57	-0.56	-0.57	-0.57	-0.56	-0.54	-0.56	-0.58	-0.61	-0.63
	G6	Press-fit	***-6.21***	***-6.17***	***-6.08***	***-5.99***	***-5.77***	***-5.55***	***-6.12***	***-6.70***	***-8.13***	***-9.56***	**-11.21**	**-12.86**
		Threaded	-3.57	-3.54	-3.48	-3.42	-3.26	-3.09	-4.08	***-5.08***	***-6.38***	***-7.67***	***-8.73***	***-9.80***
		LPOFS	***-6.19***	***-6.16***	***-6.14***	***-6.12***	***-6.07***	***-6.02***	***-6.13***	***-6.24***	***-6.88***	***-7.53***	***-8.17***	***-8.81***
	G7	Press-fit	1.92	0.28	-2.52	***-5.32***	***-8.87***	**-12.42**	**-14.45**	*-16.48*	*-16.74*	*-17.00*	*-17.10*	*-17.19*
		Threaded	4.48	3.22	0.88	-1.47	-4.60	***-7.73***	***-9.36***	**-10.99**	**-11.16**	**-11.34**	**-11.41**	**-11.48**
		LPOFS	-1.28	-1.27	-1.89	-2.52	-3.71	-4.91	***-5.85***	***-6.80***	***-6.97***	***-7.15***	***-7.33***	***-7.50***
	G8	Press-fit	*-15.44*	*-16.61*	*-16.80*	*-16.99*	*-17.00*	*-17.00*	*-17.00*	*-17.00*	*-17.00*	*-17.00*	*-17.00*	*-17.00*
		Threaded	***-9.12***	**-10.19**	**-10.39**	**-10.58**	**-10.58**	**-10.58**	**-10.58**	**-10.58**	**-10.58**	**-10.59**	**-10.59**	**-10.60**
		LPOFS	-3.34	-4.30	-4.61	-4.93	-4.99	***-5.05***	-4.97	-4.89	-4.85	-4.81	-4.77	-4.73
	G9	Press-fit	*-18.24*	*-18.25*	*-18.26*	*-18.26*	*-18.25*	*-18.25*	*-18.25*	*-18.25*	*-18.25*	*-18.25*	*-18.25*	*-18.25*
		Threaded	*-24.23*	*-24.20*	*-24.21*	*-24.23*	*-24.22*	*-24.22*	*-24.22*	*-24.22*	*-24.22*	*-24.23*	*-24.22*	*-24.21*
		LPOFS	***-6.74***	***-6.73***	***-6.72***	***-6.70***	***-6.69***	***-6.69***	***-6.69***	***-6.69***	***-6.68***	***-6.68***	***-6.67***	***-6.66***

Italic: over -15%; bold: -10% to -15%; bold-italic: -5% to -10%; underline: 0% to -5%; normal-regular: bone mass increase.

## Data Availability

No data were used to support this study.

## Abbreviations

DSA: Direct skeletal attachment
GZ: Gruen zone
LBE: Load bearing exercises
LPOFS: Limb Prosthesis Osseointegration Fixation System
OPRA: Osseointegrated Prostheses for the Rehabilitation of Amputees.
